# Dexmedetomidine suppresses sevoflurane anesthesia-induced neuroinflammation through activation of the PI3K/Akt/mTOR pathway

**DOI:** 10.1186/s12871-019-0808-5

**Published:** 2019-07-27

**Authors:** Nan Wang, Mingyu Wang

**Affiliations:** Department of Anesthesiology, Cancer Hospital of China Medical University, Liaoning Cancer Hospital & Institute, Dalian Medical University Clinical Oncology College, Shenyang, 110042 Liaoning China

**Keywords:** Dexmedetomidine, Sevoflurane, Neuroinflammation, PI3K/Akt/mTOR pathway

## Abstract

**Background:**

Sevoflurane, an inhalational general anesthetic, has become one of the most widely used inhalational anesthetics in surgery. However, previous studies have found that sevoflurane anesthesia can trigger an inflammatory response, resulting in secondary damage. Dexmedetomidine (DEX), a highly-selective α adrenergic receptor agonist, is widely used as an anesthetic adjuvant in the clinic. In this study we investigated whether DEX was able to suppress sevoflurane-induced neuroinflammation.

**Methods:**

The aim was to determine the mechanism of action of the suppressive effect of DEX using a rat model. Rats were randomly divided into a control group (*n* = 10), low-dose sevoflurane group (L-Sev; *n* = 10), high-dose sevoflurane group (H-Sev; n = 10), vehicle group (n = 10), DEX group (n = 10) and DEX + LY294002 (a specific inhibitor of PI3K) group (n = 10). The rats in vehicle, DEX and DEX + LY294002 groups were in the presence of high-dose sevoflurane exposure. Western blotting was used to measure the expression of proinflammatory cytokines (IL-6, IL-8, TNF-α) and the activity level of the phosphatidylinositol 3-hydroxy kinase/protein kinase B/mammalian target of rapamycin (PI3K/Akt/mTOR) pathway.

**Results:**

We found that sevoflurane anesthesia induced an increase in the levels of pro-inflammatory cytokines, while decreasing activation of the PI3K/Akt/mTOR pathway in both the cortex and hippocampus of rats. Treatment with DEX reduced pro-inflammatory cytokine levels and prevented inactivation of the PI3K/Akt/mTOR pathway. Moreover, LY294002, an inhibitor of the PI3K/Akt/mTOR pathway, reduced the anti-inflammatory activity of DEX.

**Conclusions:**

These data suggest that the PI3K/Akt/mTOR pathway contributes to sevoflurane-induced neuroinflammation and that activation of PI3K/Akt/mTOR signaling by DEX could help reduce the neuroinflammatory effects of sevoflurane.

## Background

In recent years the inhalational anesthetic sevoflurane has replaced other volatile anesthetics due to its quick induction, stable maintenance, rapid revival and because the irritation it causes is mild [1]. However, recent evidence has shown that sevoflurane can lead to pathophysiological alterations of the brain during the recovery period, such as neuronal apoptosis, tau aggresomes, abnormal discharge of neurons, and neuroinflammation, which cause neurodegenerative changes in the development of the mammalian brain [2–6]. Exposure to anesthesia in childhood may lead to adverse neurodevelopmental outcomes in children [7]. Despite evidence linking inhalational anesthesia to neurodegenerative effects, it is still considered safer than other methods of anesthesia, and more than 3 million children undergo inhaled anesthesia every year [8, 9]. Therefore, there is an urgent need to find anesthetic adjuvants that reduce the neurotoxicity of inhaled anesthetics.

Dexmedetomidine (DEX), a selective α2-adrenoceptor agonist, is used intravenously as a sedative or adjuvant of local anesthetics used in peripheral nerve blocks, where it prolongs the duration of sensory and motor blockades without causing toxicity [10–12].In addition, DEX is able to cross the blood brain barrier and stimulate α2-adrenoceptors centrally and has been shown to exert a neuroprotective effect [13]. Wang et al. found that treatment with DEX could inhibit the expression of inflammatory cytokines and their mediators, resulting in reduced focal cerebral ischemia-reperfusion injury in rats. These data suggest that inhibition of the nuclear factor-κB pathway (NF-κB) may be a mechanism underlying the neuroprotective action of DEX [14]. Treatment with DEX after subarachnoid haemorrhage (SAH) attenuated SAH-induced early brain injury, partially through suppression of the toll like receptor 4 (TLR4)/NF-κB pathway and the NLRP3 inflammasome [15]. Additionally, DEX has been shown to improve post-operative cognitive dysfunction in aging mice by inhibition of the hippocampal inflammatory response and reduction of neuronal apoptosis [16]. Taken together, these data suggest that DEX has anti-inflammatory properties in the central nervous system (CNS).

Given the inflammation induced by sevoflurane and the anti-inflammatory properties of DEX, we aimed to ascertain whether DEX could provide protection from sevoflurane anesthesia-induced inflammation in the CNS and ascertain whether these protective effects were closely associated with the phosphatidylinositol 3-hydroxy kinase/protein kinase B/mammalian target of rapamycin (PI3K/Akt/mTOR) pathway.

## Methods

### Animals

Fifty healthy Sprague-Dawley (SD) rats, weighing 150–200 g were purchased from the Animal Experiment Center of the Institute of Radiation Medicine of the Chinese Academy of Medical Sciences, China. The rats were fed food and water under specific pathogen-free conditions and housed in a 12 h light/dark cycle at 22–24 °C. All animal procedures were approved by the Institute of Radiation Medicine of the Chinese Academy of Medical Sciences and conducted in accordance with the ethical principles for Experiments on Animals, in addition to international standards.

### Groups and treatments

Rats were randomly divided into six groups: control group (*n* = 10), low-dose sevoflurane group (L-Sev; n = 10), high-dose sevoflurane group (H-Sev; n = 10), vehicle group (n = 10), DEX group (n = 10) and DEX + LY294002 (a specific inhibitor of PI3K) group (n = 10). All rats were placed in an anesthesia induction chamber and received anesthesia using an inhalation machine. Oxygen concentration and anesthesia doses were continuously monitored. The control group was treated with 60% O_2_ for 2 h, the L-Sev group with 1.5% sevoflurane inhalation for 2 h and the remaining groups (H-Sev group, vehicle group, DEX group and DEX + LY294002 group) were treated with 3% sevoflurane inhalation for 2 h [17]. One hour prior to sevoflurane treatment, the vehicle group received an intraperitoneal injection of saline, the DEX group received an intraperitoneal injection of 4 μg/kg DEX [18] and the DEX + LY294002 group received an intraperitoneal injection of 4 μg/kg DEX and intracerebroventricular injection of 25 μg/5 μl LY294002 (Sigma-Aldrich Chemical Company, USA) [19]. The time at which anesthesia commenced was when sevoflurane concentration had reached a maximum for each group. Gas flow in the anesthesia chamber was maintained at a rate of 4 L/min.

### Tissue preparation

At the end of the experiment, rats were sacrificed under anesthesia using 50 mg/kg sodium pentobarbital by intraperitoneal injection. The brain tissue was removed and a subset of brain samples used to prepare 10 μm sections for use in immunofluorescence staining. The remaining brains were placed on Aluminum foil and the cortical and the hippocampal tissues separated then stored at − 80 °C for Western blot analysis.

### Western blot analysis

Cortical and hippocampal tissue samples were lysed and total protein extracted using whole-cell protein extraction kits (Beyotime, China) in accordance with the manufacturer’s protocols. A bicinchoninic acid (BCA) protein quantification kit (Beyotime, China) was used to measure protein concentration in the samples. Proteins were separated using 10% SDS-PAGE then transferred to polyvinylidene difluoride (PVDF) membranes. The membranes were blocked using 2% BSA in TBST at room temperature for 2 h. Membranes were then incubated with primary antibodies overnight at 4 °C. The primary antibodies used in our study included rabbit anti-IL-6 (1:1000, Abcam, USA), IL-8 (1:1000, Abcam, USA), TNFα (1:1000, Abcam, USA), PI3K (1:1000, CST, USA), p-PI3K (1:1000, CST, USA), Akt (1:1000, CST, USA), p-Akt (1:1000, CST, USA), mTOR (1:1000, CST, USA), p-mTOR (1:1000, CST, USA) and GAPDH (1:4000, Proteintech, USA). Following staining with a primary antibody, three TBST washes were preformed and the membranes were then incubated with a horseradish peroxidase-conjugated goat anti-rabbit IgG secondary antibody (1:4000, Proteintech, USA) for 2 h at room temperature. PVDF membranes were then washed three times with TBST and developed using an ECL Plus reagent. The optical density of each protein was analyzed using Image J software (National Institutes of Health, USA) and was standardized to GAPDH or corresponding total protein.

### Statistical analysis

All values are presented as means ± SD. Comparisons between groups were conducted using t-tests and one-way ANOVA followed by a post hoc Tukey test. Differences were considered statistically significant at a level of *P* < 0.05.

## Results

### Sevoflurane induced neuroinflammation in rats

**Fig. 1:**
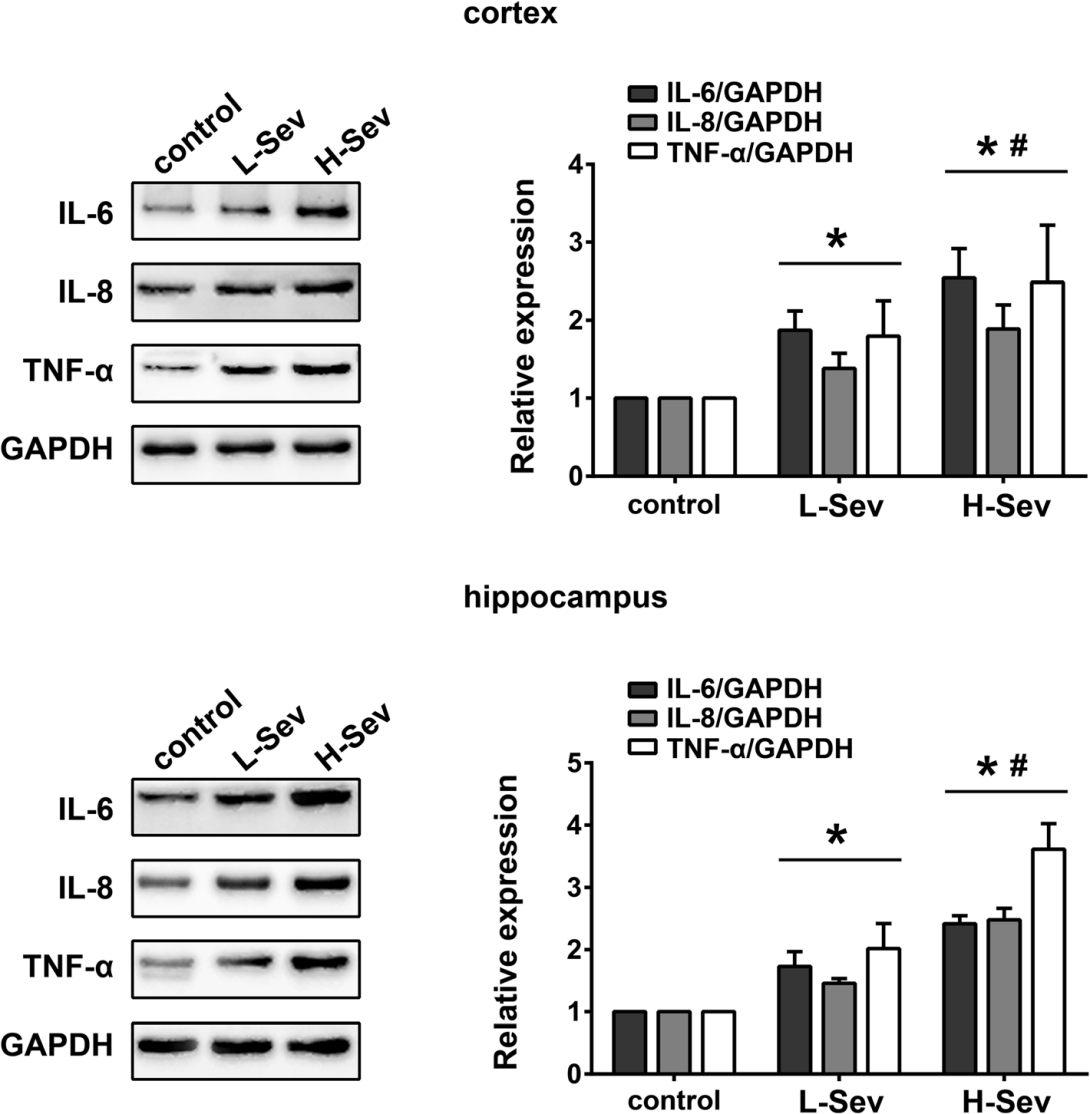
Sevoflurane treatment increases inflammatory markers in the hippocampus. Expression levels of IL-6, IL-8 and TNF-α in the cortex and hippocampus of rats in the control, L-Sev and H-Sev groups were measured by Western blotting (mean ± SD, *n* = 5 per group, one-way ANOVA). *p < 0.05 vs control group, #p < 0.05 vs L-Sev group

To determine the effect of sevoflurane on neuroinflammation, expression levels of the inflammatory markers IL-6, IL-8 and TNF-α were measured in the cortex and hippocampus of rats in the control, L-Sev and H-Sev groups by Western blotting. IL-6, IL-8 and TNF-α expression increased significantly in the cortex and hippocampus after low or high-dose sevoflurane anesthesia compared with that of the control group (Fig. 1, *p* < 0.05). Moreover, the high-dose sevoflurane group exhibited significantly higher levels of IL-6, IL-8 and TNF-α than the low-dose group, indicating that the induction of inflammation was dose-dependent (Fig. 1, *p* < 0.05).

### Sevoflurane inhibited the PI3K/Akt/mTOR pathway in rats

**Fig. 2:**
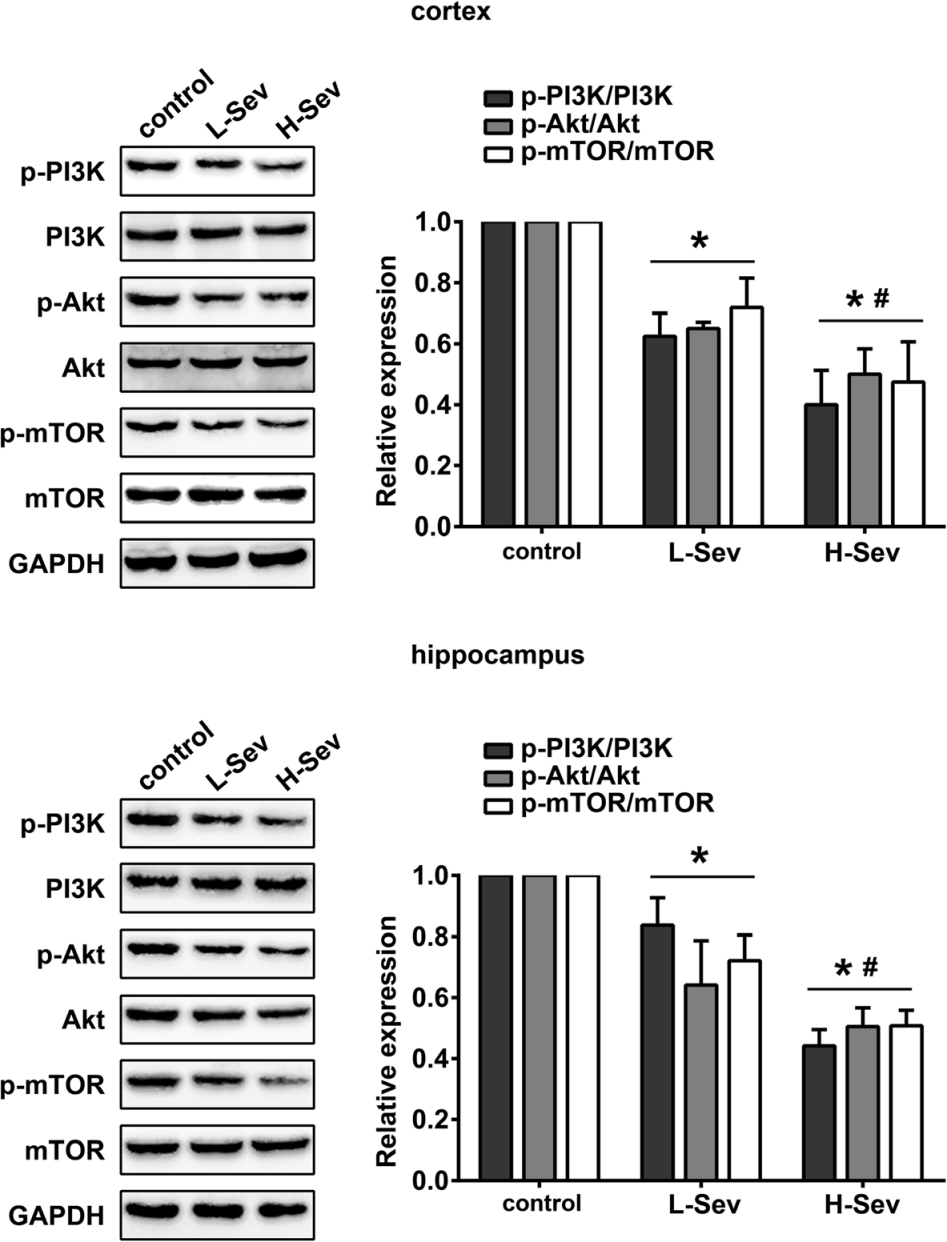
Sevoflurane inhibited the PI3K/Akt/mTOR pathway in rats. Cortical and hippocampal levels of PI3K, p-PI3K, Akt, p-Akt, mTOR and p-mTOR of rats in the control, L-Sev and H-Sev groups were measured by Western blotting (mean ± SD, *n* = 5 per group, one-way ANOVA). *p < 0.05 vs control group, #*p* < 0.05 vs L-Sev group

The levels of PI3K, Akt and mTOR expression were assayed, in addition to their phosphorylation status, in the cortex and hippocampus of rats from the control, L-Sev, and H-Sev groups in order to assess the effect of sevoflurane anesthesia on PI3K/Akt/mTOR signaling. As shown in Fig. 2, both low and high-dose sevoflurane treatment groups exhibited significantly decreased levels of PI3K/Akt/mTOR pathway phosphorylation (*p* < 0.05). Moreover, the level of PI3K/AKT/mTOR pathway activity was significantly lower in the cortex and hippocampus after high-dose sevoflurane anesthesia compared with that in the L-Sev group (Fig. 2, *p* < 0.05).

### Dexmedetomidine suppressed sevoflurane-induced neuroinflammation in rats

**Fig. 3:**
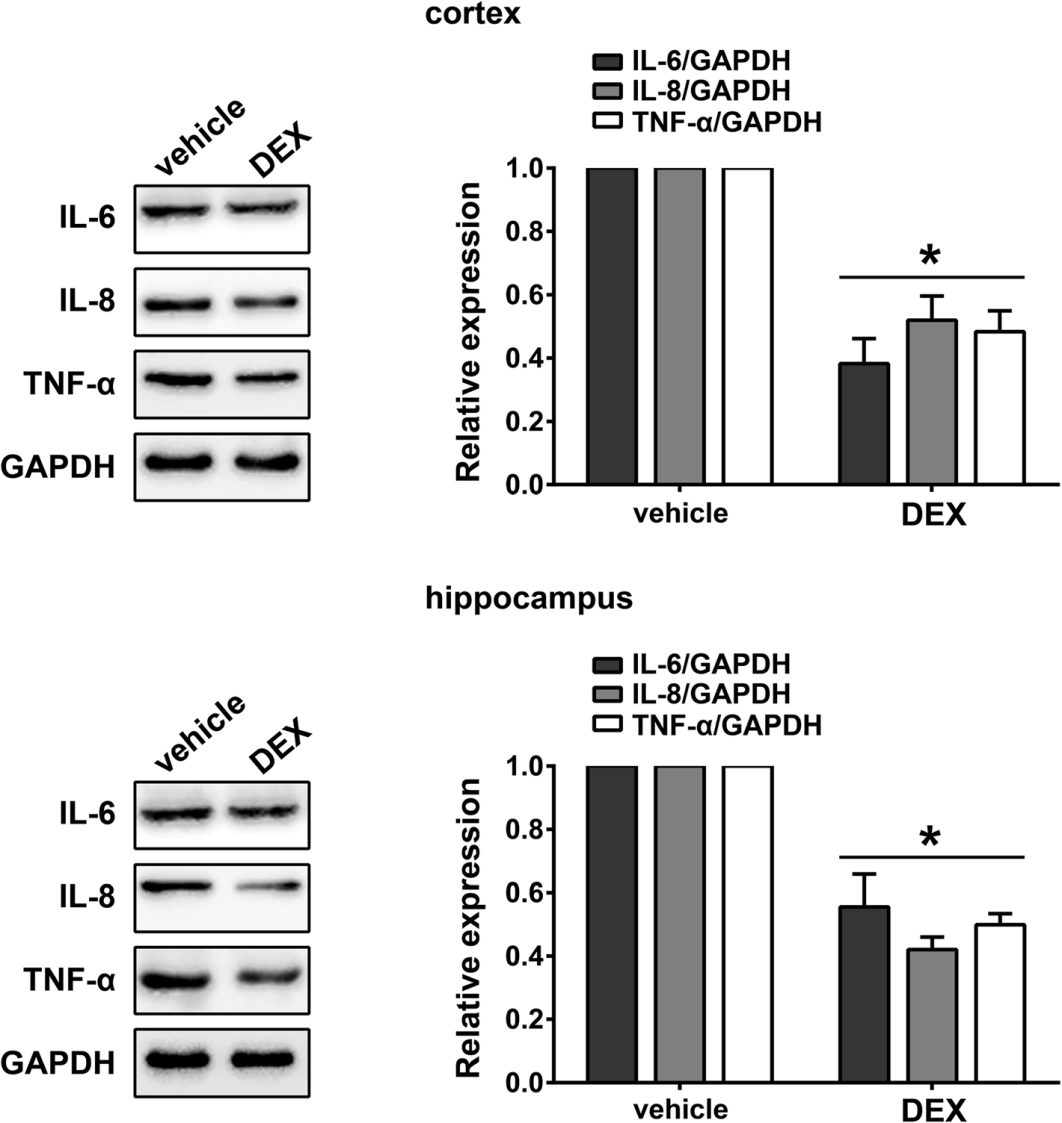
Dexmedetomidine suppressed sevoflurane-induced neuroinflammation in rats Western blot measurements of IL-6, IL-8 and TNF- α from cortical and hippocampal samples of DEX and vehicle-treated rats (mean ± SD, *n* = 5 per group, t test). *p < 0.05 vs vehicle group

To determine if dexmedetomidine could reduce neuroinflammation induced by sevoflurane anesthesia, the levels of IL-6, IL-8 and TNF-α were measured in the cortex and hippocampus of rats in the vehicle and DEX groups. Rats treated with DEX exhibited decreased levels of IL-6, IL-8 and TNF-α compared with the vehicle control group, suggesting that DEX can inhibit sevoflurane anesthesia-induced neuroinflammation (Fig. 3, *p* < 0.05).

### Dexmedetomidine activated the PI3K/Akt/mTOR pathway in sevoflurane-treated rats

**Fig. 4:**
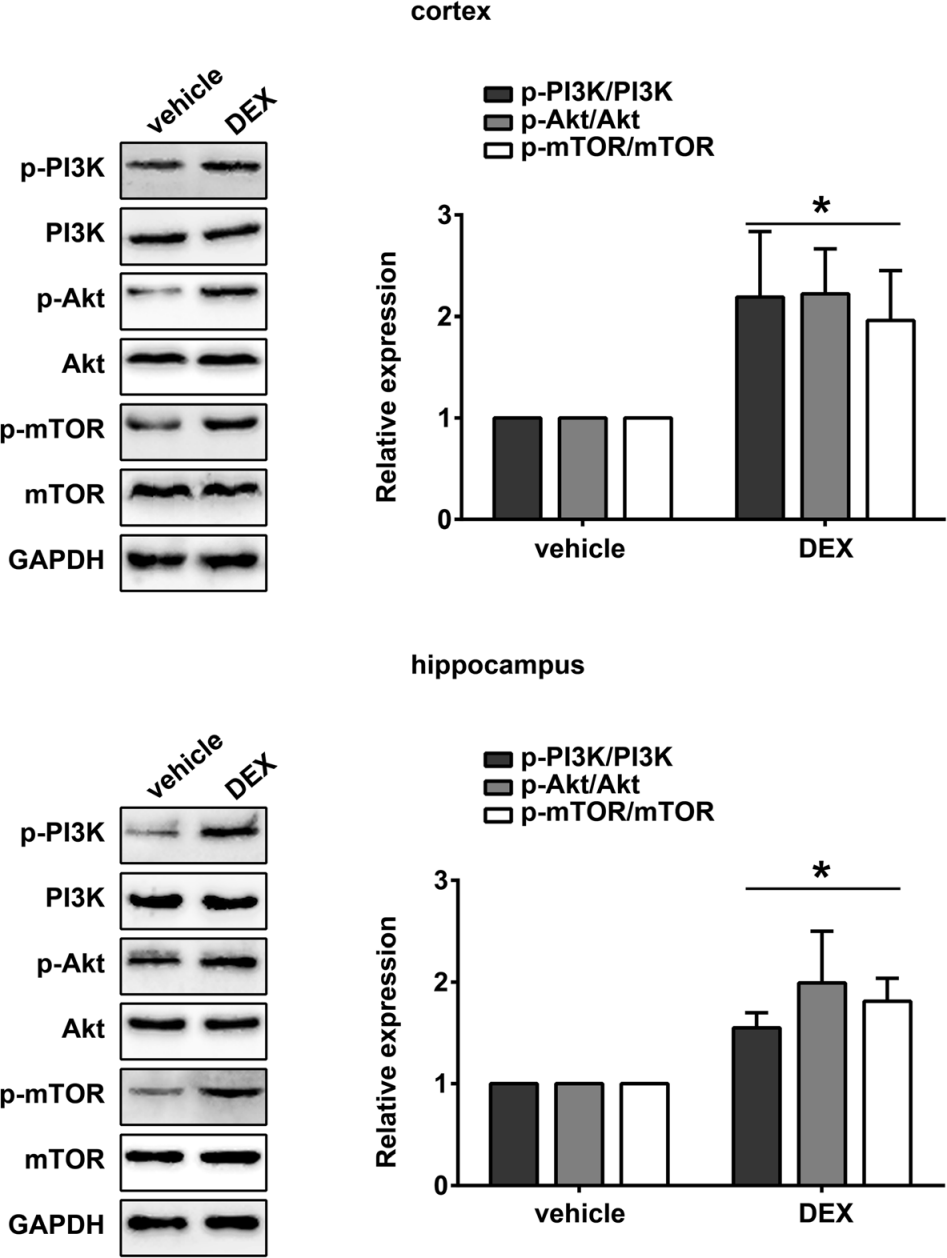
Dexmedetomidine activated the PI3K/Akt/mTOR pathway in sevoflurane-treated rats Images and quantification of Western blot measurements of PI3K, p-PI3K, Akt, p-Akt, mTOR and p-mTOR in the cortex and hippocampus of rats in the vehicle and DEX groups (mean ± SD, *n* = 5 per group, t test). *p < 0.05 vs vehicle group

Using Western blotting, we assayed the effects of DEX on PI3K/Akt/mTOR pathway activation. As shown in Fig. 4, the levels of PI3K, Akt and mTOR phosphorylation increased significantly in the cortex and hippocampus in the DEX group compared with the vehicle group, indicating that DEX could activate the PI3K/Akt/mTOR pathway under sevoflurane anesthesia (p < 0.05).

### Blockade of the PI3K/Akt/mTOR pathway reduced the anti-inflammatory activity of dexmedetomidine

**Fig. 5:**
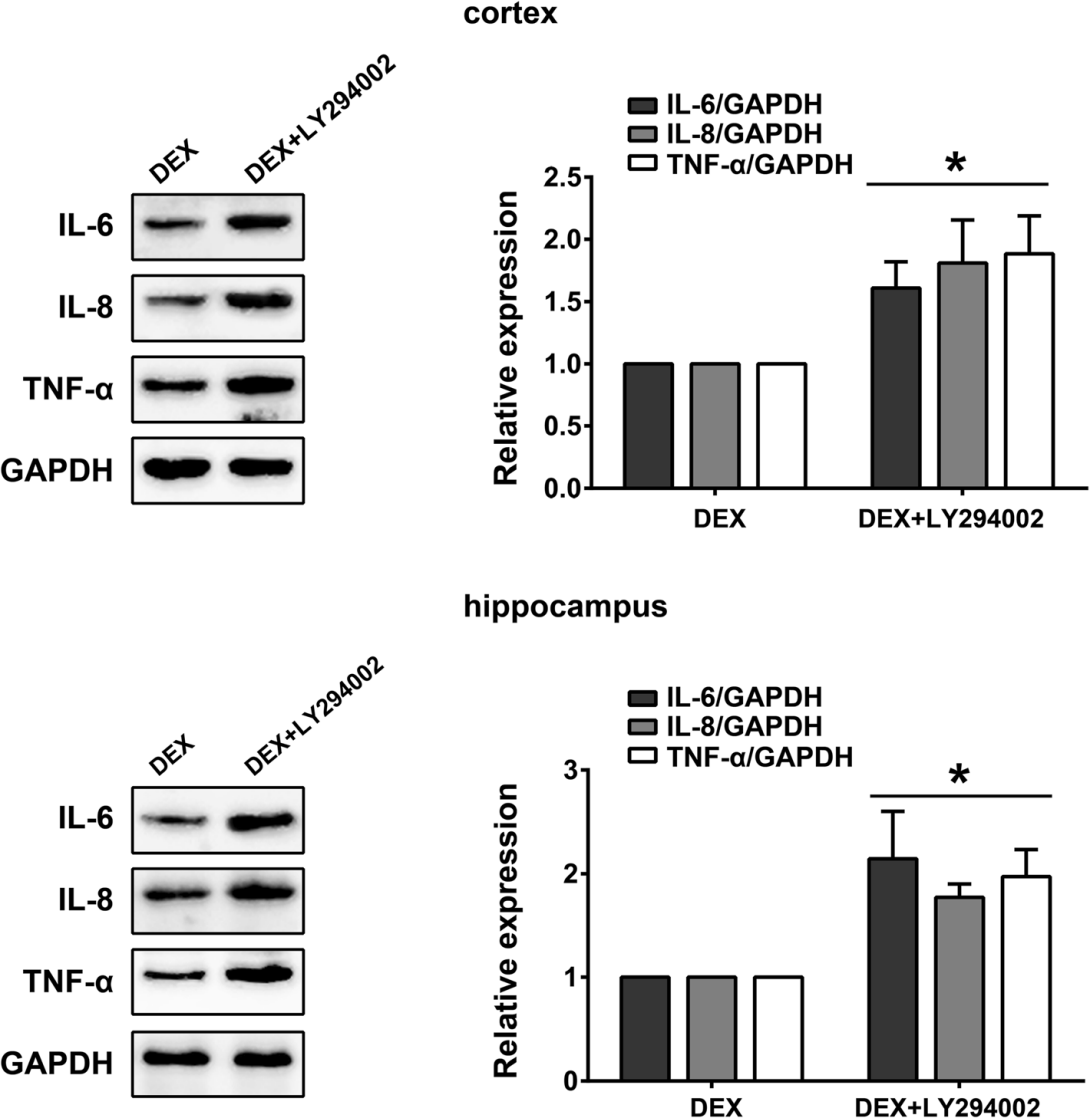
Blockade of the PI3K/Akt/mTOR pathway reduced the anti-inflammatory activity of dexmedetomidine Western blot measurements of IL-6, IL-8 and TNF- α from cortical and hippocampal samples of rats in the DEX and DEX + LY294002 groups (mean ± SD, *n* = 5 per group, t test). *p < 0.05 vs DEX group

Finally, to determine whether the PI3K/Akt/mTOR pathway is involved in the anti-inflammatory effects of DEX, rats were treated with DEX in the presence or absence of LY294002 and the expression of IL-6, IL-8 and TNF-α in the cortex and hippocampus assessed by Western blot analysis. As shown in Fig. 5, the levels of IL-6, IL-8 and TNF-α increased significantly in the cortex and hippocampus in the DEX+LY294002 group compared with the DEX group, indicating that LY294002 reduced the anti-inflammatory activity of DEX under sevoflurane anesthesia (*p* < 0.05).

## Discussion

In the present study, we observed that sevoflurane anesthesia induced an increase in the expression of proinflammatory factors and a decrease in PI3K/Akt/mTOR pathway activity in both the cortex and hippocampus of rats. Additionally, we found that DEX treatment could restore PI3K/Akt/mTOR activity in rats treated with sevoflurane anesthesia and blockade of the PI3K/Akt/mTOR pathway reduced the anti-inflammatory activity of DEX. Thus, we propose that DEX can suppress sevoflurane anesthesia-induced neuroinflammation by modulating PI3K/Akt/mTOR pathway activity.

Postoperative neuroinflammation is a common pathological phenomenon in the CNS, which can lead to secondary damage, such as delirium, cognitive dysfunction, Alzheimer’s disease and additional detrimental effects [20, 21]. A healthy immune response is crucial to ensure proper wound healing and repair of tissue damage, in addition to combating infection without harming the host’s own cells or tissues. In contrast, an excessive inflammatory response is harmful and can result in severe tissue damage and even death [20, 22]. Inhalational anesthetics, such as isoflurane and sevoflurane, can trigger pathological inflammatory responses during surgery. Isoflurane, which has been used since the 1980s, is metabolized slowly leading to reduced induction of anesthesia during surgery and shorter recovery times [23]. The use of sevoflurane began a decade later. It has a lower blood-gas partition coefficient than other anesthetics, leading to rapid induction of anesthesia and faster recovery times after anesthesia [24, 25]. Several decades of research have been conducted on the toxicity and side effects of these volatile anesthetics. Here, we investigated the relationship between sevoflurane anesthesia and neuroinflammation. We used low- or high-dose sevoflurane in rats, then detected the expression of proinflammatory cytokines (IL-6, IL-8, TNF-α). We confirmed that sevoflurane could increase the levels of IL-6, IL-8 and TNF-α in the cortex and hippocampus of anesthetized rats, in agreement with previous research which indicated that both isoflurane and sevoflurane were able to increase IL-6 levels via activation of NF-κB signalling [26].

The PI3K/Akt/mTOR cascade is important in mediating the release of proinflammatory cytokines [27–29]. PI3K is a ubiquitous lipid kinase which plays a crucial role in signal transduction through receptor tyrosine kinases. PI3K phosphorylates phosphatidylinositol-4,5-bis-phosphate (PIP2) to form phosphatidylinositol-4,5-tri-phosphate (PIP3); PIP3 recruits other downstream molecules, such as serine-threonine kinases, including the major effector of PI3K activation, Akt [30]. Activated Akt can then in turn activate mTOR, leading to the phosphorylation of two downstream effectors, p70 ribosomal protein S6 kinase 1 (S6K1) and eIF4E binding protein (4E-BP1), which promotes translational initiation and elongation [31–34]. In addition to its role in protein synthesis, mTOR also regulates the ubiquitin-proteasome system (UPS) [34, 35]. The UPS is an important regulator of NF-κB signaling, as the inhibitory regulator of NF-κB must be degraded by the proteasome in order for NF-κB to become active. Harston et al. suggested that inhibition of mTOR by rapamycin could increase polyubiquitination and ubiquitin-mediated degradation of IκB-α, leading to NF-κB-induced transcriptional activation. This result suggests that activation of mTOR could prevent the occurrence of inflammation through suppression of NF-κB activation [27], as NF-κB activity promotes the expression of proinflammatory cytokines [28, 29]. We observed an increase in pro-inflammatory cytokine release after treatment with sevoflurane anesthesia, and this release was accompanied by the inactivation of the PI3K/Akt/mTOR pathway, suggesting that inactivation of the PI3K/Akt/mTOR pathway may be associated with sevoflurane anesthesia-induced neuroinflammation. Thus, increasing PI3K/Akt/mTOR pathway activity may be a promising and novel therapeutic strategy against postoperative neuroinflammation.

Recent studies have suggested that DEX has anti-inflammatory properties. Chen et al., for example, found that DEX treatment could suppress retinal ischemia/reperfusion injury, and showed effective anti-inflammatory effects through inhibition of toll-like receptor 4 (TLR4)/NF-κB expression [36]. Additionally, Huang et al. also suggested that DEX could inhibit the nuclear translocation and binding activity of activated NF-κB, thus reducing the release of inflammatory cytokines [37]. Given the anti-inflammatory properties of DEX and its effects on the PI3K/Akt/mTOR signaling pathway, we hypothesized that DEX could suppress sevoflurane anesthesia-induced neuroinflammation. We found that treatment with DEX down-regulated pro-inflammatory cytokines (IL-6, IL-8 and TNF-α) and up-regulated the phosphorylation levels of PI3K/Akt/mTOR. A previous study indicated that DEX exerts an anti-inflammatory effect via the activation of PI3K/Akt/mTOR signaling in rats with traumatic brain injury [19]. In addition, blockade of the PI3K/Akt/mTOR pathway reduced the anti-inflammatory activity of DEX. Thus, we propose that DEX can ameliorate sevoflurane-induced neuroinflammation through activation of the PI3K/Akt/mTOR signaling pathway.

## Conclusions

Sevoflurane anesthesia induces neuroinflammation in the CNS and this inflammation may be the result of decreased signaling through the PI3K/Akt/mTOR pathway. The administration of DEX can reduce neuroinflammation caused by sevoflurane, providing an important reference basis for clinical anesthesia.

## Data Availability

The datasets used and/or analyzed during the current study are available from the corresponding author by reasonable request.

## Abbreviations

DEX: Dexmedetomidine
NF-κB: Nuclear factor-κB pathway
PI3K/Akt/mTOR: Phosphatidylinositol 3-hydroxy kinase/protein kinase B/mammalian target of rapamycin
PIP2: Phosphatidylinositol-4,5-bis-phosphate
PIP3: Phosphatidylinositol-4,5-tri-phosphate
S6K1: S6 kinase 1
SAH: Subarachnoid haemorrhage
TLR4: Toll like receptor 4
UPS: Ubiquitin-proteasome system
